# Training, supervision, and experience of coaches offering digital guided self-help for mental health concerns

**DOI:** 10.3389/fpsyg.2023.1217698

**Published:** 2023-11-22

**Authors:** Ellen E. Fitzsimmons-Craft, Elsa Rojas, Naira Topooco, Gavin N. Rackoff, Nur Hani Zainal, Daniel Eisenberg, Jillian Shah, Christina Desage, Denise E. Wilfley, Craig Barr Taylor, Michelle G. Newman

**Affiliations:** ^1^Department of Psychiatry, Washington University School of Medicine in St. Louis, St. Louis, MO, United States; ^2^Center for m (2) Health, Palo Alto University, Palo Alto, CA, United States; ^3^Department of Behavioral Sciences and Learning, Linköping University, Linköping, Sweden; ^4^Department of Psychology, State College, Penn State University, University Park, PA, United States; ^5^Harvard Medical School, Department of Healthcare Policy, Boston, MA, United States; ^6^Department of Health Policy and Management, Fielding School of Public Health at UCLA, Ann Arbor, MI, United States; ^7^Children and Adolescents Psychotherapy and Technology Lab, Palo Alto University, Palo Alto, CA, United States; ^8^Department of Psychiatry and Behavioral Sciences, Stanford University School of Medicine, Stanford, CA, United States

**Keywords:** guided self-help, coaches, coaching, training, digital mental health

## Abstract

Accessible, low-cost intervention options are necessary to address the rise in mental health problems among college students. Digital guided self-help, or coached, programs have been developed to provide such services, with many commercially available. As such, there are a large and growing number of individuals coaching these programs. However, an unmet need is to evaluate and assess best practices for training and supervising individuals in these positions. To this end, we describe how we recruited, trained, and supervised coaches as part of a large randomized controlled trial using a widely available digital commercial platform. Coaches were trained to provide digital guided self-help for depression, anxiety, and/or eating disorders for college students. Coaches initially attended three live training sessions over 2–3 weeks, viewed multiple training videos, and read a detailed coaching manual developed by our team. Thereafter, they attended weekly supervision. Following their term, coaches completed an exit survey to assess their supervision and training experiences. A total of 37 of 70 (53%) graduate-level student coaches completed the survey. The experience was reported as very positive (95%). In particular, the majority reported feeling well prepared, more confident, and felt they had developed useful skills for their own practice.

## 1 Introduction

Recent work suggests that half of all college students suffer from one or more mental health disorders, but relatively few receive treatment (Lipson et al., 2022). To meet the need for more accessible, lower-cost mental health services, digital guided self-help, or coached, programs have been developed. These programs have demonstrated effectiveness for numerous mental health disorders (Newman et al., 2011a,b; Olthuis et al., 2015; Karyotaki et al., 2018; Fitzsimmons-Craft et al., 2020; Taylor et al., 2021), and several companies now provide digital guided self-help programs and employ coaches. For example, SilverCloud Health is one example of a company providing these services, with numerous studies demonstrating the effectiveness of programs delivered on this platform (e.g., Richards et al., 2015; Palacios et al., 2018). The current number of individuals providing these services is unknown, but mental health coaching has been identified as a clear industry trend (Landi, 2020; Bestsennyy et al., 2021; Wood, 2021; DeAngelis, 2022; Moon, 2022). In just the third quarter of 2021, over $3.1 billion was invested in digital mental health startups (DeAngelis, 2022), many of which offer these kinds of programs. Despite the growth of this sector of mental health services, little has been written about how to best train and supervise individuals coaching digital mental health programs or about the experience of coaches who support these programs.

Unlike more open-ended psychotherapy, in coached programs, there is typically a structured, self-help intervention or set of content that individuals work through. As such, the coach’s role is to support the individual’s use of the program rather than provide traditional psychotherapy. However, coaches need to be prepared to address issues raised by users, and therefore, most coached mental health programs assume that coaches should have specific training in mental health, including working with clients, or at least on the mental health issues addressed by the intervention (Werntz et al., 2023). Furthermore, some digital guided self-help programs include real-time personal contact via phone, video, and/or SMS—activities where experience in providing psychotherapy would seem beneficial (Linardon et al., 2019). Other programs may only have coaches offer asynchronous support, for example, through written messages (Mehta et al., 2019).

Prior work has described what coaches do (e.g., Slovensky et al., 2017; Linardon et al., 2019; Phillips et al., 2019; Fitzsimmons-Craft et al., 2020; Werntz et al., 2023). However, these papers have not elaborated on the precise training activities or content of the coach training for delivering the intervention. Widely accepted competencies for asynchronous digital mental health training have also not been developed, although competencies for telepsychiatry have been suggested (Hilty et al., 2020).

Coaches should have some training in the theoretical model guiding the guided self-help program to help users optimally understand and utilize program features. For instance, in programs based on cognitive-behavioral therapy (CBT), coaches must be able to explain the theoretical model of problem maintenance and answer questions about how and why each of the skills work. They also need to be able to offer suggestions for how to apply the strategies being taught to daily life. Furthermore, they need to determine when specific strategies should be recommended. Moreover, part of coach training needs to help coaches acquire the skills to provide this feedback in written format to users.

Another issue is training coaches in the technical aspects of using the platform to provide digital guided self-help. This process is relatively straightforward, and videos and structured practice sessions can be used to provide this training. At the same time, though, supervisors of coaches in these programs must be prepared to address technical questions and problems.

A general assumption has been that training and supervision for coaches of these programs should be similar to any other training of new mental health providers, with added emphasis, perhaps, on addressing issues of particular importance to digital interventions (Richards and Richardson, 2012). Such training might include how to deal with poor adherence to the program and how to write effective but succinct feedback messages. It should also likely include review of written coach messages and feedback as appropriate. However, relatively little has been written on this topic, and to date, limited guidance is available in terms of best practices for these activities, though Lattie et al. (2019) provide helpful guidance on defining the scope and development of text-based coaching protocols for digital mental health interventions.

The main purpose of this paper was to describe how coaches were recruited, trained, and supervised to provide guided self-help for depression, anxiety, and eating disorders in college students, using a widely available digital commercial platform. A secondary goal was to assess coaches’ experience with the training and providing digital guided self-help. Together, this information can begin to provide information on best practices for training and supervising digital mental health coaches in future research, commercial endeavors, and other implementation efforts—an incredibly important undertaking given the proliferation of these services.

## 2 Methods

### 2.1 Context

The training described below was provided as part of a large randomized controlled trial. The trial was designed to test the effectiveness of a transdiagnostic, low-cost mobile mental health targeted prevention and intervention platform for depression, anxiety, and eating disorders in college students (Fitzsimmons-Craft et al., 2021). Students at 26 participating colleges and universities were sent an email inviting them to complete an online mental health screen. Those who screened positive or were at high risk for clinical depression, anxiety, and/or an eating disorder and who were not in mental health treatment were invited to participate. If interested, they then completed informed consent and were randomly assigned to a digital guided self-help intervention provided on the SilverCloud Health platform (described below) or referral to usual care available on their campus (e.g., through the student mental health center).

### 2.2 SilverCloud

The digital guided self-help program was provided on the SilverCloud Health platform. SilverCloud is a for-profit company that offers a variety of digital self-help programs for mental health concerns in both guided and unguided formats. For this study, the SilverCloud platform offered guided interventions to address depression, anxiety, and eating disorders. The interventions were derived from evidence-based, CBT-guided self-help treatments shown to be effective previously in randomized trials for depression, anxiety, and EDs (Spek et al., 2007; Andrews et al., 2010; Newman et al., 2011a; Richards and Richardson, 2012; Davies et al., 2014; Fitzsimmons-Craft et al., 2020; Taylor et al., 2021). Six to eight core modules were offered for each primary problem, which took about 20 min each. Modules included a combination of psychoeducational content and quizzes, interactive exercises, videos, and personal stories (Fitzsimmons-Craft et al., 2021). Students were offered access to the intervention for 6 months. For users with multiple problems, key modules to address comorbidities were provided after the initial content from the primary program was offered. In this way, the program was personalized to address each user’s unique needs.

### 2.3 Coaching

The coach’s role included supporting and enhancing user motivation, monitoring progress, facilitating goal setting and offering accountability, providing feedback on technique usage and encouraging practice, answering user questions, and tracking for/responding to clinical risk. In this project, communication with users was primarily via two-way, asynchronous messaging, with an optional, supplementary phone call at the beginning of the user’s time in the program to enhance goal setting. Coach messaging was done via a web-based “dashboard” and delivered to users within the environment of the program (app or web browser access). The dashboard allowed coaches to monitor multiple users at one time efficiently.

**Figure 1:**
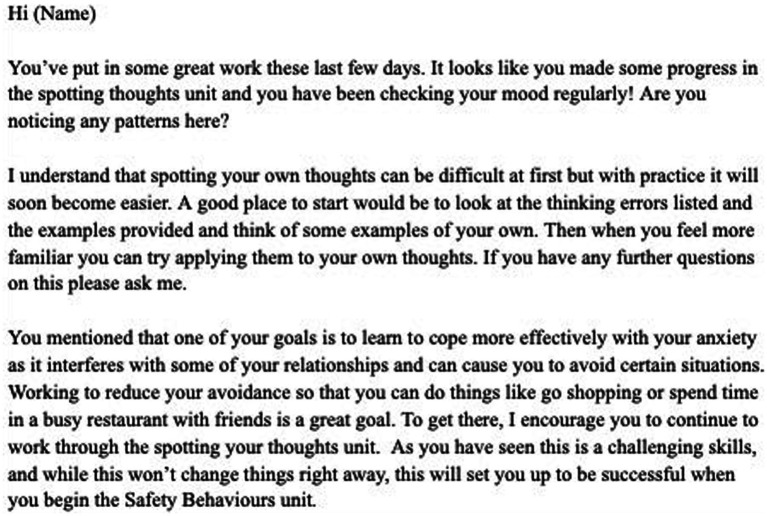
Example of sample message.

Messages were primarily unscripted to allow for personalization, although editable templates guided some common situations (especially risk-related ones, including standard referral messages when a user endorsed suicidal ideation). An example of sample messages from the manual can be seen in Figure 1.

The goals of reinforcing key intervention tenets, supporting the individualized application of techniques, and promoting meaningful engagement with content guided messaging, which included the following: feedback on technique completion, helping users apply program content to personal goals, commenting on observed symptom changes, and engaging in motivational interviewing techniques such as open-ended questioning. Coaches had participants’ baseline screen results so they could personalize intervention and help users apply skills learned to comorbid problems. If the coach felt the mobile program could not adequately address the user’s needs, they were referred to appropriate campus/community resources. Referrals to outside services most often occurred when users expressed symptoms related to comorbidities the program was unable to address (e.g., substance use or those expressing significant distress related to past trauma) or when a user was expressing significant or increased suicidal ideation. Users with clinical problems who did not demonstrate improvement in their symptoms by 6 weeks into the intervention were also provided with a referral, per the study protocol. Users continued to have access to SilverCloud even after receiving a referral.

### 2.4 Training

#### 2.4.1 Selection

Selected coaches were required to be in a master’s, PhD, or PsyD mental health training program. They were recruited from several specific counseling, PhD, and PsyD programs, along with a general recruitment message sent out through social media, listservs, etc. Prospective coaches needed to make a one-year commitment to the study and complete all required training. This included training sessions on the basics of CBT as applied to each of the primary diagnoses of interest for the study (depression, anxiety, eating disorders), training related to the general study protocol, and training on digital coaching.

#### 2.4.2 Initial coach training activities

Once an individual was accepted as a coach, they were sent an onboarding email, invited to create a SilverCloud account, and given access to a Box folder with all of the training videos and a detailed coach training manual. This onboarding email outlined the administrative tasks requested (e.g., CITI training) and provided a recommended order to begin reviewing the information on their own before the training sessions. Coaches were encouraged to start by watching a coach overview training video, which provided a general overview of the study aims, basic information on using SilverCloud, and an overview of the study protocol (other videos included training on CBT for depression, anxiety, and eating disorders, and coach expectations and tools). Coaches were also given access to the training manual, a 132-page document that provided detailed information related to implementing coaching (available at https://www.m2health.paloaltou.edu/resources). The training manual provided an overview of the program, discussed guidelines and the coaches role, the purpose of using symptoms to monitor treatment, how to address engagement, make referrals, address crises, and write reviews, and also included overviews of the various programs. The manual also included step-by-step instructions on how to use the SilverCloud platform. In addition, it described a set of best practices to guide coaching activities derived from those used by SilverCloud and from our previous studies (Fitzsimmons-Craft et al., 2020; see Table 1). These best practices were frequently referred to during training, and were categorized by frequency, with certain practices expected every 2–3 messages to a user or from “time to time” and others being expected in more than 90% of messages or “most times.”

**Table 1:** Coach best practice rubric.

Target coach aims to meet at the beginning and from time to time: Yes/ No
Manages or clarifies user roles and expectations and reviews sessions
Demonstrates interest in the user as a person
Helps user clarify or specify primary concerns or goals
Encourages use of SilverCloud (SC)
Target coach aims to meet for most review sessions (compulsory): Yes/ No
Reflects progress related to identified problems – anxiety, depression, ED
Uses open-ended questions to guide treatment
Reinforces positive steps (even if minuscule)
Supports user autonomy
Target coach aims to meet for some review sessions (optional): Yes/ No
Connects to CBT formulation (thoughts-feelings-actions)
Brainstorms solutions or alternatives
Asks any other open-ended questions
Answers any concerns users raise about SC modules
Validates or normalizes user’s challenges
Reminds the user to put the things they are learning into practice (self-efficacy promotion)
Makes appropriate and personalized recommendation content and tools
use - giving a clear rationale for why (set homework)
Gives clear deadlines for the completion of tasks - conscious of tone and
avoiding being too directive (set homework)
Things that coach should not do: Yes/ No
Reinforces bad behavior or mindsets
Over-shares personal information
Interprets underlying motives
Gives direct and specific advice in patronizing, condescending, or other unhelpful ways

Following the initial training, coaches were asked to view a series of training videos that described the study and reviewed basic CBT approaches for depression, anxiety, and eating disorders. At this time, coaches also received access to the SilverCloud platform and were encouraged to explore features such as bonus training resources provided by the company, the ability to engage with the program as a mock user, and other resources that provided information on CBT basics and specific techniques (e.g., cognitive restructuring). Coaches were asked to spend several hours familiarizing themselves with the various programs and getting a feel for how SilverCloud looked to users.

#### 2.4.3 Live (over zoom) training sessions

Coaches were expected to attend 3 live training sessions and received an email outlining the homework and expectations for these trainings prior to their first session. During the training sessions, the coaches were assigned to act as a coach for one of the previously created mock user accounts, allowing all coaches a chance to practice writing reviews before being assigned their first participant. They were provided individual feedback on the practice review, and these were reviewed in the final training session as well. Overall, coaches were told that it would take 10–15 h to complete the training activities, which they were encouraged to complete in 2–3 weeks.

#### 2.4.4 Supervision

Ongoing supervision of coaches while working with real users was provided by senior clinicians. Before each supervision meeting, coaches completed a supervision log, a brief online form where they reported their weekly hours and listed any concerns or questions they wanted to discuss in supervision. This form allowed supervisors to prepare ahead of time to address concerns. Additionally, each week, supervisors briefly reviewed each coach’s work in SilverCloud. This mainly entailed checking to see that reviews were sent out promptly, and every 2–3 weeks, each coach’s work was “spot-checked” to review general protocol compliance. Concerns identified were discussed with individual coaches and reviewed in supervision.

#### 2.4.5 Supervision session goals/principles

Supervision sessions occurred weekly during the semester and bi-weekly over school holidays and summer when new users were not brought on. Each supervision session was 45–60 min long. Supervision sessions began with a review of any administrative issues (e.g., upcoming launches of the program at a new university), and, when indicated, a review of any feedback from the adherence team (see below), followed by a discussion of any issues identified on the supervision logs. Most importantly, the supervisor reviewed issues and cases of interest, and, every quarter, reviewed general issues like how to respond to suicidal ideation or to handle a user who reported a trauma. The sessions encouraged coaches to focus on the protocol, including using outcome monitoring to know when to make referrals and to provide feedback to users. Other common issues often covered included maintaining one’s role as a coach (vs. functioning as a therapist), encouraging user engagement with the program, and normalizing that limited engagement with the program can be typical and often had nothing to do with the coach personally, a common coach worry. At a minimum, coaches reported on their own users individually in the supervision sessions at least every 2–3 weeks.

#### 2.4.6 Adherence team feedback

An adherence team reviewed about 5% of all messages sent by coaches to users, using a rubric that mapped on to the best practices (Table 1). This information was then sent to the supervisors, who provided individual feedback outside of regular supervision by email. Deidentified issues and problems of general interest or importance raised by the adherence team were brought by the supervisors to the supervision sessions.

#### 2.4.7 Support group

Coaches were provided access to a support group offered on Slack to connect with one another and ask questions, without supervisors present.

### 2.5 Evaluation

#### 2.5.1 Recruitment

Individuals who had served as coaches as part of this randomized controlled trial were sent an anonymous survey to assess their training, supervision, and coaching experience. The email invitation provided a brief description of the study’s purpose (“The survey is designed to both help us in future studies and to understand better the experiences of people doing online coaching more generally”). Two reminders were sent out by email to participants to complete the survey. Participants were offered a $10 Amazon gift card for study participation. To receive the gift card, they completed a linked survey to maintain their anonymity. Human subject permission was obtained from the Washington University in St Louis IRB to collect anonymous data.

#### 2.5.2 Survey

The questionnaire created for evaluation was modeled based on resources and examples of questionnaires concerning the evaluation of training elements in higher education that were publicly available through university and college websites (e.g., Stanford University, University of Michigan). Based on this review, questions were designed in four thematic areas appropriate to the evaluation of training elements and practical curriculum that the coaches of the project were undergoing: (1) Background and prerequisites (previous experience with therapy and digital interventions), (2) Overall assessment (rating the experience as a whole and most and least liked aspects), (3) Process (assessment of the implementation of the training and coach practicum respectively; e.g., the usefulness of each training component, perceived own effort, difficulty and self-motivation in training/coaching, and potential areas for improvement), and (4) Outcomes (perceived relevance of the experience, e.g., the extent to which it resulted in skills of relevance, motivation to be trained/return to practice in digital mental health in the future). The full survey is provided in Appendix A.

## 3 Results

### 3.1 Participants

Overall, 75 coaches were trained. Two coaches missed several meetings/responsibilities and resigned. Forty coaches (53% of the sample, 40/75) returned the survey. Of these, 3 completed only the initial part of the survey and were not included. Of the 37 who completed the entire survey the mean age of respondents was 31 (*SD* = 7.67, range = 23–53) (see Table 2 for all demographics).

**Table 2:** Demographic characteristics (Total *N* = 37).

	*N* (%)
Gender	
**Woman**	**30 (81)**
Man	6 (16)
Other	1 (3)
Age	
**20–29 years old**	**17 (46)**
30–39	13 (35)
40–50	4 (11)
51+	1 (0)
Decline to state	2 (1)
Race/Ethnicity	
Asian	9 (24)
African American/Black	1 (3)
**White**	**21 (57)**
Mixed	2 (5)
Did not respond	4 (11)
Status as clinician/trainee	
**Master’s student**	**22 (60)**
PhD student	9 (24)
PsyD student	3 (8)
Other	2 (5)
Did not respond	1 (3)

### 3.2 Coaching experience

The overall coaching experience was reported as very positive (4.13 on a 1–5 Scale, poor to excellent). Most respondents (>75%) felt all aspects of the training experience were very or extremely useful, with the one exception being the support group. Although offered to all coaches, only 15/37 availed themselves of this opportunity (see Table 3 for all results).

**Table 3:** Coach ratings of training components.

Component	N^*^	Usefulness rating, *n* (% of those who responded to the item & n):			
		Extremely	Very	Slightly	Not
Coaching manual	37	23 (62.2%)	11 (29.7%)	3 (8.1%)	0 (0%)
Individual feedback from adherence team on areas of review	30	10 (33.3%)	14 (47.7%)	20.0 (6%)	0 (0%)
General feedback from adherence team to group	31	10 (32.3%)	15 (48.3%)	6 (19.3%)	0 (0%)
Mock reviews	37	17 (46.0%)	11 (29.7%)	9 (24.3%)	0 (0%)
Supervision	37	18 (48.7%)	11 (29.7%)	7 (18.9%)	1 (2.7%)
Live Zoom training	29	11 (37.9%)	12 (41.4%)	6 (20.7%)	0 (0%)
Depression training video	36	14 (38.9%)	13 (36.1%)	8 (22.2%)	1 (2.8%)
Anxiety training video	36	14 (38.9%)	13 (36.1%)	8 (22.8%)	1 (2.8%)
Eating disorders training video	37	14 (37.8%)	13 (32.4%)	9 (24.3%)	1 (2.7%)
Support group	15	7 (46.7%)	2 (13.3%)	6 (40%)	0 (0%)

^*^N = number who rated usefulness of that item.

In addition, almost all participants (35/37, 94.6%) felt that the training sufficiently prepared them for their coaching assignment. Nearly all participants (36/37, 97.3%) strongly agreed or agreed that the training helped them feel confident in delivering guided self-help, and 91.8% (34/37) reported that they developed knowledge/skills useful for their professional practice. Furthermore, 91.8% (34/37) of coaches strongly agreed or agreed that they enjoyed the coaching experience.

Most coaches (>70%) felt the experience made them more competitive in achieving their career goals. Of interest, 73.0% (27/37) felt that digital mental health training practice should be included as a course in their training programs, and 94.6% (35/37) felt that such experience should be “counted” as official clinical training hours for their programs (see Table 3 for all results).

Respondents provided open-ended comments on their perception of the positive aspects of their coaching experience. These included working with students, the SilverCloud platform, supervision, and learning more about and implementing CBT. The most negative aspects were the low student engagement and the tedium of sending out reminder messages to student users to engage. Coaches reported that they would prefer to spend less than 50% of their time working as a guided self-help coach in the future for similar programs.

## 4 Discussion

The goal of this paper was to describe training and supervision of coaches for a CBT-based, digital guided self-help program for mental health problems and to report on coaches’ experience. Coaches participated in initial training activities, live training sessions, and ongoing supervision with senior clinicians, with the ultimate goal being to train them to be able to provide support, motivation, and feedback to users through two-way, asynchronous messaging within the program platform. Overall, coaches were very satisfied with the training and felt confident about providing the intervention. All components of the training were rated positively by almost all trainees. Overall, the data from the coaches’ surveys suggested that this approach successfully provided training to help trainees feel confident in providing the program (see Tables 4, 5).

**Table 4:** Coach ratings of training experience.

Component	N^*^	Strongly Agree	Agree	Neither agree or disagree	Disagree	Strongly disagree
The training sufficiently prepared me for my coach assignment	37	17 (45.9%)	18 (48.6%)	1 (2.7%)	1 (2.7%)	0 (0%)
The training motivated me to do my best	37	17 (45.9%)	18 (48.6%)	3 (8.1%)	1 (2.7%)	0 (0%)
The feedback I received impacted my learning	37	17 (45.9%)	18 (48.6%)	2 (5.4%)	0 (0%)	0 (0%)
The coaching experience suited my level of knowledge and skills	37	17 (45.9%)	11 (29.7%)	0 (0%)	2 (5.4%)	0 (0%)
The coaching tasks were well organized	37	18 (48.6%)	11 (29.7%)	2 (5.4%)	0 (0%)	0 (0%)
I had a clear understanding of what was expected of me as a coach	37	19 (51.4%)	16 (43.2%)	1 (2.7%)	1 (2.7%)	0 (0%)
I enjoyed being a coach	36	19 (52.8%)	15 (41.7%)	0 (0%)	2 (5.6%)	0 (0%)
Takeaways from training and coaching						
I feel confident in my competency to work as a guided self-help coach for a similar program	37	29 (78.4%)	7 (18.9%)	0 (0%)	1 (2.7%)	0 (0%)
I developed knowledge/skills useful for my (future) professional practice	37	27 (73.0%)	7 (18.9%)	1 (2.7%)	1 (2.7%)	0 (0%)
The experience has made me more competitive in achieving my career goals	37	14 (37.8%)	14 (37.8%)	6 (16.2%)	2 (5.4%)	1 (2.7%)
Digital mental health training-practice should be included as a course in my program	37	15 (40.5%)	12 (32.4%)	5 (13.5%)	3 (8.1%)	2 (5.4%)
The hours of supervision and coaching should count towards hours need for internship, placement, etc.	36	24 (66.7%)	11 (30.6%)	0 (0%)	0 (0%)	1 (2.7%)

^*^N = number who rated the usefulness of that item. ^**^% of those who rated the item.

**Table 5:** Training sessions.

Training session 1:
*Prior to training:* Coaches sign up for SilverCloud and watch training videos in the Box folder. *The goal of this session:* Supervisor to answer any questions about the study protocol and getting started. This includes ensuring each coach has been able to create their SilverCloud account, answering questions about how to complete CITI training, and allowing for time to discuss questions that arose from watching the training videos. *Homework to be completed after this session:* Coach creates a mock user account in SilverCloud and uses the account for several hours over one week.
Training Session 2:
*Prior to training:* Coach completes any remaining training videos and reviews the study manual. *The goal of this session:* Discussion of the review process and provision of tips and helpful guidance. Addressing general questions that came up as coaches continued to familiarize themselves with the study and with SilverCloud. *Homework to be completed after the session:* Each coach was assigned a mock client and asked to write a practice review.
Training Session 3:
*Prior to training:* Coach has completed all training videos/items and has submitted practice review. *The goal of this session:* Supervisor goes over practice reviews and discusses strategies for improving. Time is also allocated for reviewing steps to writing the reviews and reviewing best practices.

Because this effort was implemented as a part of a research project, coaches were also provided weekly supervision, and a random sample of their coaching messages was assessed for adherence to the program. These data will be reported in detail in a future publication. Most coaches (81%) reported liking the feedback from the adherence team. Quality assurance measures of adherence to the protocol are essential for research studies but can also play a useful role in training, as evidenced by the coaches’ positive ratings of this aspect of the approach. Technology is emerging to automatically analyze written transcripts in a number of ways, including adherence, so that this information could be made available to coaches and supervisors in a less labor-intensive and cost-effective way (Jiang et al., 2021). As such, in the future, such a practice could be more readily incorporated into even very large-scale dissemination efforts.

Whereas our main goal was to provide excellent training and supervision for coaches in a research study, we also wanted to develop a model that could be used for training individuals to serve as coaches for similar programs more generally or even outside of the context of a research study. Of note, 73% of coaches felt that training in digital approaches should be included in mental health training programs, and most importantly, all but one coach felt that coaching hours should be counted as official clinical hours in mental health training programs. This is important as currently there is a high degree of variability in whether such hours “count” across various mental health graduate degree programs. Organizations that certify mental health training programs, such as the American Psychological Association, the National Association of Social Workers, and the American Association for Marriage and Family Therapy, should adopt policies officially supporting training in various digital mental health approaches and “counting” the hours providing intervention in these approaches (Pote et al., 2021). Coaches in this study reported, on average, that they would like to work 30% of the time as a guided self-help coach in the future if reimbursed. Since the pandemic began, use of telehealth has skyrocketed, with usage ~38 times higher than it was pre-COVID (Bestsennyy et al., 2021). Therapists have also been found to now be more accepting of telehealth, with 64% reporting an authentic experience with their clients via telehealth (Petersen et al., 2020). However, what is needed now is further acknowledgment of and training in a wide range of digital technologies to support individuals with mental health concerns (not just telehealth but also digital guided self-help, etc.; Goncharova, 2021; Zielasek et al., 2022).

The model we provided is purely digital, but in the future, it is very possible our approach could be “blended” with more traditional psychotherapy (either in person or via telehealth), and it is anticipated that such approaches will become even more widely employed in the future (Baumeister et al., 2020, 2021). Much support and acceptance for blended therapy has been shown; in a randomized control trial, psychotherapists in the intervention group who were shown a video on blended therapy had a small to medium effect on performance expectancy, effort expectancy, and facilitating conditions (Baumeister et al., 2020). Teletherapy can easily be added to the type of digital program in which therapists in this study were trained.

### 4.1 Limitations

Although individuals can acquire many important skills via the training approach in this study, there are also a number of limitations to this as a treatment modality. Engagement with digital guided self-help programs is typically short, with high rates of individuals logging into the intervention just a handful of times or less. Trainees reported that the greatest frustration of the intervention/training was dealing with the lack of engagement. Furthermore, digital interventions are often much less interactive than face-to-face psychotherapy or teletherapy, as communication is asynchronous and relies on participant engagement in the program and response to messages. As such, a major focus of the coaches’ approach is to foster self-management of issues. There may also have been selection bias in terms of the coaches who chose to complete the feedback survey having more positive experiences. Finally,

the structured format of the surveys may have constrained the coaches’ reports of their experiences and lead to oversimplification. In addition, respondents’ accounts may be susceptible to recall bias.

## 5 Conclusion

Coaches of a digital guided self-help program for mental health problems reported both positive and helpful feedback about their training and supervision experiences. Overall, training enabled them to feel prepared for their work as a coach and to report confidence in their ability to support users. This work could serve as a model for future efforts to train and supervise coaches to offer digital guided self-help and also supports the need to offer this type of training in mental health programs in the future, “count” these hours, and support efforts for such services to be reimbursed moving forward.

## Data availability statement

The raw data supporting the conclusions of this article will be made available by the authors, without undue reservation.

## Ethics statement

The studies involving human participants were reviewed and approved by Washington University IRB (IRB ID# 202202085). The studies were conducted in accordance with the local legislation and institutional requirements. The participants provided their written informed consent to participate in this study.

## Author contributions

EF-C, ER, NT, NZ, GR, CT, and MN contributed to the conception and design of the coach training and this study. JS organized the database. EF-C, DE, DW, CT, and MN obtained funding. EF-C, ER, NT, NZ, GR, JS, CD, CT, and MN drafted the manuscript. All authors contributed to the article and approved the submitted version.
