# Supplementary material for: Training, supervision, and experience of coaches offering digital guided self-help for mental health concerns
Source: Front Psychol. 2023 Nov 22;14:1217698. doi: 10.3389/fpsyg.2023.1217698 (PMC10698738; doi:10.3389/fpsyg.2023.1217698)
Supplement: Supplementary file 1 [file Data_Sheet_1.docx]

Supplementary Material

Training, Supervision, and Experience of Coaches Offering Digital Guided Self-help for Mental Health Concerns

Ellen E. Fitzsimmons-Craft ^1*^, Elsa Rojas^2^, Naira Topooco^2, 3^, Gavin Rackoff^4^, Hani Zainal, Daniel Eisenberg^5^, Jillian Shah^1^, Christina Desage^6^, Denise E. Wilfley^1^, Craig Barr Taylor^2 ,7^, Michelle Newman^4^

*** Correspondence:**Ellen E. Fitzsimmons-Craft
fitzsimmonse@wustl.edu

# Supplementary Data

**Appendix A**

**Survey Questionnaire**

**Consent:**

Welcome to the iAIM EDU Coach study!

We greatly appreciate you taking the time to participate. Please review the consent form on the next page. If you have any questions, please reach out to our study coordinators at mcallewaert@wustl.edu.

ONLINE PARTICIPANT INFORMATION STATEMENT

We invite you to participate in a research study being conducted by investigators from Washington University in St. Louis. In this study you will be asked to complete a brief survey on your experience as a coach for the iAIM edu study. The survey is anonymous. You will receive a $10 Amazon gift card to complete this study. In order to receive payment you will be taken to another survey where you will be asked to enter your name, email address where you want the gift card sent to and your social security number (SSN). If you do not wish to share your SSN you will be able to enter your address and citizenship status. This information will only be used for payment purposes as required by Washington University in St Louis. This information will be kept separately from your study data. If you have questions for the research team, please contact Marie-Laure Firebaugh at 314- 286- 0253 or you may contact the Human Research Protection Office at 1- (800)-438-0445 or hrpo@wustl.edu. Thank you very much for your consideration of this research study.

Please click below if you wish to print a copy of this consent form: Consent Form

Have you read the consent form, and do you agree to participate in the study as described in the consent form?

[ ] Yes, I have read the consent form, and I agree to participate in this study.

[ ] No, I decline participation in this study

Thank you for considering this survey.

We appreciate your contributions as a coach!

**Questionnaire**

**Demographics:**

1. What is your age? Please enter a whole number only - no letters or words ___________.
2. What is your current institution?

- PAU
- PSU
- WashU
- Other (please specify): __________.

1. What is your gender identity?

- Cisgender male
- Cisgender female
- Another gender identity

1. What is your race?

- American Indian or Alaska Native
- Asian
- Black of African American
- White
- Native Hawaiian or Pacific Islander
- Multiracial
- Unknown or prefer to not answer

1. What is the highest level of education you have completed? (Please specify your major):

- Bachelor's Degree in: __________.
- Master's Degree in: __________.
- PhD. In: __________.
- Psy.D in: __________.
- Postdoc in: __________.
- Other (please specify): __________.

1. What degree did you pursue while coaching for iAIM?

- Master's in: _________.
- PhD. in: _________.
- Psy.D in: _________.
- Postdoc in: _________.
- Other (please specify): _________.

**Background:**

1. Prior to AIM:

Not at all Very little Somewhat To a great extent

Did you have any experience

conducting therapy? [ ] [ ] [ ] [ ]

Did you have any theoretical

training in CBT? [ ] [ ] [ ] [ ]

Did you have any experience

with digital coaching? [ ] [ ] [ ] [ ]

1. If you have experience conducting therapy prior to iAIM, what would you consider your primary theoretical orientation?

- No prior experience
- Behavioral
- Cognitive behavior
- Eclectic/Integrative
- Humanistic/Existential
- Interpersonal
- Psychodynamic/Psychoanalytic
- System
- Other, please specify: ___________

1. Which of the following best represents your career goals? Select all that apply:

- Clinician / Mental Health Professional
- Research
- Other, please specify: ___________

**Overall Assessment:**

1. Please answer the following questions on your overall experience with iAIM:

Very Poor Poor Average Good Excellent

How would you rate your

overall experience with iAIM? [ ] [ ] [ ] [ ] [ ]

1. Which aspects of the study did you like the most:______________________.
2. Which aspects of the study did you like the least: ______________________.

**Process-Training Experience:**

1. Please rate how useful each of the training components in iAIM were:

Extremely Very Slightly Not useful No basis

useful useful useful at all for judgment

iAIM Coaching manual

(PDF) [ ] [ ] [ ] [ ] [ ]

Individual email feedback

from the adherence team

on areas of your reviews [ ] [ ] [ ] [ ] [ ]

General email feedback

from the adherence team

to the coach group [ ] [ ] [ ] [ ] [ ]

Mock reviews of another

coach [ ] [ ] [ ] [ ] [ ]

Supervision sessions [ ] [ ] [ ] [ ] [ ]

Live Zoom Training

sessions [ ] [ ] [ ] [ ] [ ]

Required training video

– Body Eating

Concerns [ ] [ ] [ ] [ ] [ ]

Required training video

– Depression [ ] [ ] [ ] [ ] [ ]

Required training video

– Anxiety [ ] [ ] [ ] [ ] [ ]

Support group in which

coaches could discuss

coaching difficulties

without supervisor

evaluation [ ] [ ] [ ] [ ] [ ]

Other 1 (please

specify):_______ [ ] [ ] [ ] [ ] [ ]

Other 2 (please

specify):________ [ ] [ ] [ ] [ ] [ ]

1. Please rate the statements about the training you received in iAIM:

Strongly Agree Neither agree Disagree Strongly

agree or disagree disagree

The training sufficiently

prepared me for my

coach assignment [ ] [ ] [ ] [ ] [ ]

The training motivated

me to do my best [ ] [ ] [ ] [ ] [ ]

The feedback I received

impacted my learning [ ] [ ] [ ] [ ] [ ]

1. What can we change to improve this aspect?: __________________________.

**Process - Coaching Experience:**

1. Please rate the following statements about your coaching experience:

Strongly Agree Neither agree Disagree Strongly

agree or disagree disagree

The coaching experience

suited my level of

knowledge and skills [ ] [ ] [ ] [ ] [ ]

The coaching tasks

were well organized [ ] [ ] [ ] [ ] [ ]

I had a clear

understanding of what

was expected of me as [ ] [ ] [ ] [ ] [ ]

a coach

I enjoy being a coach [ ] [ ] [ ] [ ] [ ]

1. What can we change to improve this aspect?: __________________________.
2. While you provided coaching in iAIM – how many hours per week on average did you spend on your coach assignment (including continuous supervision/training), the whole number of hours? ____________________________________________________.
3. How would you rate your coach workload?

Too little About right Too much

The coach workload [ ] [ ] [ ]

Thank you so much for your answers so far! 😊

**Outcomes/Results:**

1. Please rate the statements about your takeaways from iAIM:

Strongly Somewhat Neither agree Disagree Strongly N/A

agree agree or disagree disagree

I feel confident in my

competency to work as

a guided self-help [ ] [ ] [ ] [ ] [ ] [ ]

coach for a similar

program in the

future

I developed

knowledge/skills

useful for my [ ] [ ] [ ] [ ] [ ] [ ]

(future)

professional

practice

The experience

has made me more

competitive in [ ] [ ] [ ] [ ] [ ] [ ]

achieving my

career goals

Digital mental

health training-

practice like iAIM

should be included [ ] [ ] [ ] [ ] [ ] [ ]

as a course in my

program

The hours of

supervision and

coaching should

count towards [ ] [ ] [ ] [ ] [ ] [ ]

hours needed for

internship,

placement etc.

1. What can we change to improve this aspect?: ___________________________.
2. Following iAIM, how has your motivation to work with digital mental health changed?

Much Lower About the Higher Much

lower same higher

My motivation to work with

digital mental health has [ ] [ ] [ ] [ ] [ ]

become:

22.) What can we change to improve this aspect?: ___________________________.

23.) If reimbursed or if you could be paid specifically for this work – to what extent

would you like to work as a guided self-help coach for similar programs in the

future?

- 0% of my hours
- 10%
- 30%
- 40%
- 50% of my hours
- 60%
- 70%
- 80%
- 90%
- 100% of my hours
- I don’t plan to work as a Mental Health professional

24.) For what reasons would you most likely return to coach digital self-help programs

in the future? ______________________________________________________.

We appreciate all of the feedback you've provided so far!

**Credibility Expectancy Questionnaire:**

Please answer the following questions on your experience with the Silvercloud treatment offered in iAIM

25.) From your perspective as coach:

Not at all-1 Somewhat-5 Very-9

1 2 3 4 5 6 7 8 9

How logical does

the Silvercloud

treatment offered to [ ] [ ] [ ] [ ] [ ] [ ] [ ] [ ] [ ]

clients seem to

you?

How successful do

you think the [ ] [ ] [ ] [ ] [ ] [ ] [ ] [ ] [ ]

Silvercloud treatment

will be in reducing

client’s mental health

symptoms?

How confident would

you be in

recommending the

Silvercloud treatment [ ] [ ] [ ] [ ] [ ] [ ] [ ] [ ] [ ]

to a friend who

experiences problems

similar problems to

clients in iAIM?

26.) Following the Silvercloud treatment, how much improvement in symptoms in clients’ symptoms do you think will occur?

0 10 20 30 40 50 60 70 80 90 100

Improvement (%)

27.) From your perspective as a coach – How effectively do you think the Silvercloud

programs offered to clients were?

Extremely Very Slightly Not effective No Basis

effective effective effective at all for judgment

Space from

Depression [ ] [ ] [ ] [ ] [ ]

Space for Anxiety [ ] [ ] [ ] [ ] [ ]

Space from Body

and Eating Concerns [ ] [ ] [ ] [ ] [ ]

28.) Please use this space to share additional perspectives and recommendations, or

thoughts you may have based on your experience providing coaching for a digital

program and your clients’ use of these programs: __________________________.

End

Thank you so much for sharing your perspectives! Your anonymous response will help guide future research and implementation initiatives aimed at improving mental health among college students.

As a thank you for your participation, we would like to offer you a $10 electronic gift card.

If you would like to be compensated, please click here to provide your SSN or contact and residency information.

To provide you with this gift card, we need to collect your Social Security Number

(SSN). This is a requirement for tax-reporting purposes at Washington University in St. Louis, the institution that is awarding the prizes. If you are not willing to provide us with your SSN, but still wish to collect your gift card, you have the right to provide us with your contact information, as well as indicate whether you are a U.S. citizen, permanent resident, or a non-resident alien, instead. Again, this information will only be used for tax-reporting purposes. PRIVACY NOTE. Your SSN or contact & residency information will be collected in a separate survey and will never be linked to your anonymous response data that is used for our study. Like all data affiliated with this study, your personal information is kept completely confidential and is encrypted and protected at a secure research site.

Would you like to receive a payment for completing this survey?

- Yes, you will be redirected to the payment survey
- No
